# Performance-Based Usability of Medication Adherence Technologies Among Older Adults With Diverse Capabilities: Quantitative Study

**DOI:** 10.2196/88398

**Published:** 2026-07-13

**Authors:** Bincy Baby, Ghada Elba, SooMin Park, Imra Hudani, Rishabh Sharma, Kirk Patterson, Annette McKinnon, Sara J T Guilcher, Feng Chang, Linda Lee, Catherine Burns, Dagmar Hajducek, Ryan H Griffin, Tejal Patel

**Affiliations:** 1 School of Pharmacy, University of Waterloo, 10 Victoria St S A, Waterloo, ON, N2G 1C5, Canada, 1 (519) 888-4499 ext 21337; 2 Patient Advisors Network, Toronto, ON, Canada; 3 Leslie Dan Faculty of Pharmacy, University of Toronto, Toronto, ON, Canada; 4 Department of Family Medicine, McMaster University, Hamilton, ON, Canada; 5 Systems Design Engineering, Faculty of Engineering, University of Waterloo, Waterloo, ON, Canada; 6 National Research Council Canada, Otttawa, ON, Canada; 7 Schlegel-UW Research Institute for Aging, Waterloo, ON, Canada

**Keywords:** usability, older adults, medication adherence technology, functional abilities, medication management

## Abstract

**Background:**

Medication adherence technologies (MATs) offer innovative solutions to support older adults in managing complex medication regimens, yet usability challenges can prevent their successful use. Older adults often face cognitive, physical, sensory, motivational, and environmental barriers, which can influence how they interact with these devices. Therefore, performance-based usability testing is essential for identifying usability issues.

**Objective:**

This study aimed to evaluate the performance-based usability and user experience of 13 MATs among older adults with diverse capabilities.

**Methods:**

A prospective mixed methods design was used, with 96 participants aged 60 years and older testing smart and electronic MATs using cognitive walkthroughs and predefined usability tasks without formal training to simulate first-use conditions. This manuscript focuses specifically on the performance-based usability metrics collected as part of the mixed methods study. Validated tools, including the Self-Medication Assessment Tool (SMAT), Daily Living Tasks Dependent on Vision (DLTV), Whisper Test, Self-Efficacy for Appropriate Medication Use Scale (SEAMS), and Martin and Park Environmental Demands (MPED) Questionnaire, were used to assess individual barriers. Poisson generalized estimating equations (GEE) was used to determine predictors of unassisted task success and error rates while accounting for repeated observations.

**Results:**

A total of 96 participants (mean age 75.1, SD 7.7, range 61‐95 y) were included, with 37.5% (36/96) male and 62.5% (60/96) female. Poisson GEE models identified significant predictors of unassisted task success rate, including cognitive score (*P*<.001), physical score (*P*<.001), vision score (*P*<.001), motivational score (*P*<.001), and lower environmental busyness (*P*=.049). Female sex was associated with a 9% lower success rate (*P*=.002). Predictors of total error rate included age (*P*=.02), sex (*P*≤.001), physical score (*P*≤.001), vision score (*P*=.004), DLTV vision score (*P*=.01), MPED routine score (environmental; *P*=.05), and interaction between physical score and sex (*P*=.004).

**Conclusions:**

Both device design and user characteristics strongly influence the usability of MATs. Performance-based evaluations provide actionable findings for user-centered design and selection, guiding manufacturers and health care providers toward developing and choosing accessible technologies that could improve medication adherence among older adults.

## Introduction

In Canada, the senior population is increasing rapidly and is expected to make up nearly a quarter of the total population by 2040 [1]. As individuals age, their likelihood of developing chronic conditions significantly increases [1-5]. Multimorbidity is associated with polypharmacy, resulting in medication regimen complexity [1-6]. The complexity of such regimens, coupled with age-related cognitive and physical declines, poses significant barriers to medication self-management and adherence [7,8].

For older adults, the ability to manage their own medications is vital to maintaining independence and safety [7-9]. Defined as the ability to organize, take, and adhere to prescribed treatments, self-management requires cognitive functions, such as memory and comprehension, as well as physical capabilities like hand dexterity and visual acuity [8]. Cognitive limitations, such as memory loss and reduced problem-solving abilities, can prevent adherence to prescribed regimens [10,11]. Physical challenges, including arthritis or reduced hand strength, make tasks like opening medication containers difficult [10,12]. Sensory impairments, such as vision loss, can prevent individuals from distinguishing between medications or reading labels accurately [10,13]. Additionally, motivational factors such as low health literacy or reduced confidence, and environmental challenges, such as limited caregiver support or disorganized home environments, can further complicate medication self-management [10,14-16]. These issues often lead to medication nonadherence, resulting in adverse drug events, preventable hospitalizations, and higher health care costs [17,18].

Medication adherence technologies (MATs) offer innovative solutions to support older adults in managing their medications effectively [19,20]. These technologies make use of automation, connectivity, organization, and reminder functions to simplify medication regimens, reduce errors, and improve adherence [19-22]. For example, automated dispensing and reminder systems can reduce cognitive load and support individuals with memory-related challenges, while organized medication compartments and guided workflows may lessen reliance on visual interpretation of labels. While MATs can help address some of the functional challenges encountered by older adults by reducing cognitive load, simplifying medication routines, enabling automated medication dispensing, and facilitating communication between patients, caregivers, and health care providers, they do not eliminate these underlying impairments [21-24]. In fact, these impairments may also influence how effectively individuals interact with these technologies [21,23,25]. Cognitive impairments can limit the ability to understand or operate these devices, while physical difficulties, such as arthritis or tremors, can interfere with interaction with small buttons or complex mechanisms [10,25]. Sensory impairments, including vision and hearing loss, can make it difficult to engage with screens or auditory prompts [10,25]. Moreover, low technology literacy and resistance to adopting unfamiliar tools further restrict MATs' adoption [10,14,25]. The cost of these technologies and limitations in home environments, such as poor internet connectivity, also pose significant barriers [16,26].

The usability of MATs plays an important role in their effectiveness, efficiency, and user satisfaction [27]. Usability is defined as “the extent to which a product can be used by specified users to achieve specified goals with effectiveness, efficiency, and satisfaction in a specified context of use” [28]. For older adults, poor usability can result in medication errors, frustration, device abandonment, and even preventable hospitalizations [27,29]. Usability metrics are vital for evaluating how well users interact with MATs [27]. These metrics can be divided into perception-based and performance-based categories [30-32]. Perception-based metrics focus on users’ subjective satisfaction and feelings about a product’s usability, often captured through standardized tools such as the System Usability Scale (SUS) or the Usefulness, Satisfaction, and Ease of Use (USE) questionnaire [30-32]. Performance-based metrics, in contrast, focus on quantifiable outcomes of user interactions, offering a more objective assessment of usability [30-32]. Key performance metrics include task success, time-on-task, error rates, efficiency, and learnability [30-33]. Unassisted task success evaluates users’ ability to complete specific tasks independently, with lower success rates often reflecting both user-related factors (eg, cognitive, physical, and sensory abilities) as well as potential design-related challenges [30-33]. For example, Ligons et al in a study investigating how cognitive status affects the usability of a medication delivery device (EMMA), found that cognitive impairments reduced success rates for older adults from 69% for individuals with Mini-Mental State Examination (MMSE≥24) to 34.7% for individuals with MMSE<24 [29]. Time-on-task measures the time required to complete tasks, where longer times often indicate inefficiencies in design or difficulties arising from user limitations [30-33]. Error rates, categorized into “slips” and “mistakes,” highlight areas where both interface complexity and user challenges may contribute to confusion [30-33]. Efficiency reflects the effort required to complete tasks, considering both the number of steps and the time spent, and may be influenced by the interaction between device design and user abilities. Learnability assesses how quickly users adapt to a device over time, which can depend on both the intuitiveness of the design and the user’s cognitive and functional capacity [30-33].

By focusing on performance-based metrics, it is possible to pinpoint specific usability problems, such as which features or tasks are most challenging for older adults based on their individual limitations [33]. Barriers to usability, such as confusing interfaces or physically demanding mechanisms, can be identified and addressed to improve functionality, reduce frustration, and improve adoption rates [30-33]. Therefore, the objective of this research is to evaluate the usability and user experience (UX) of 13 different MATs among older adults with diverse physical, cognitive, sensory, motivational, and environmental abilities by examining various performance-based metrics such as unassisted task success rates, total error rates, total task completion time, and efficiency for unassisted tasks. Through comprehensive usability testing, this study aims to identify the design strengths and weaknesses of these devices for older adults with varying limitations, offering actionable recommendations to improve MATs’ usability, functionality, and adoption.

## Methods

### Study Design

This research used a prospective mixed methods design, integrating both quantitative and qualitative approaches. Quantitative methods included cognitive walkthroughs (refer to Study Procedure section), measurement of various performance-based usability metrics, and assessment of perception-based usability using standardized usability questionnaires. This paper focuses specifically on the performance-based metrics collected in this study.

### Recruitment, Eligibility, and Sample Size

Participants aged 60 years and older who were able and willing to provide informed consent were included in the study. Individuals who could not speak or read English were excluded. The sample size for the quantitative analyses was determined by applying Green’s formula to ensure sufficient power for regression models, including 6 independent variables (physical, cognitive, vision, hearing, motivational, and environmental limitations). Based on the formula n>50+8 m (where “m” represents the number of independent variables), the recommended sample size was calculated as 98 [34]. In addition, considerations from usability research informed the recruitment strategy. Previous work by Nielsen and Landauer [35] suggests that testing with a small number of users (n=5 approximately) can identify the majority (80%‐85%) of usability issues [35,36]. Accordingly, for each device, we aimed to recruit at least 5 participants representing each type of functional limitation to ensure adequate identification of usability challenges across diverse user groups.

A total of 13 devices were selected for testing. A Microsoft Excel tracking sheet was used to monitor device testing across participants and impairment types, supporting balanced representation across devices. Recruitment targeted a diverse participant pool using convenience, purposive, and snowball sampling methods, with outreach conducted through community organizations, senior fairs, professional networks, and other initiatives. Data collection occurred between June 2023 and June 2024 among older adult residents of Ontario.

### MATs Tested

A total of 13 MATs were tested, including three smart devices (SMs) and 10 electronic devices. Electronic MATs typically include features such as multiple compartments, visual or audible alarms, and timers that remind patients when it is time to take their medications [21]. While these devices may operate electronically, they generally do not connect to external networks or systems. Smart MATs incorporate advanced features such as connectivity and automaticity [22]. Connectivity refers to the ability of these devices to connect to other systems or networks using technologies such as Wi-Fi, Bluetooth, or mobile data [22]. This allows smart MATs to synchronize data with health care providers’ systems, send reminders to patients via smartphones or other connected devices, and alert caregivers or family members if a dose is missed. Automaticity refers to the device’s ability to perform functions without continuous manual input from the user [22]. This may include automatically dispensing medications at scheduled times, recording the timing and quantity of doses taken, and adjusting medication delivery based on real-time data from connected health monitoring devices [22].

These 13 devices were selected for their diversity of features in order to capture the widest range of functionalities and were guided by a taxonomy of MATs based on device features [37]. Participants tested between 1 and 7 devices across multiple sessions, depending on their preference and availability, with some requiring 2 visits to complete the testing. To ensure consistency and minimize potential bias, each device was tested by at least 5 participants representing different types of limitations. Participants were not provided with any formal training; instead, they were instructed to rely solely on the instructional materials provided with the devices, mimicking real-world independent learning scenarios as much as possible in a laboratory setting.

Table 1 provides details of the MATs tested along with their assigned codes, which will be used to refer to the various devices in this paper.

**Table 1.** List of MATs^a^ tested.

Device code	Device name	Manufacturer
APD^b^ 001	MedReady 1700 Automated Medication Dispenser	MedReady Inc
APD 002	GMS Med-e-lert Automatic Pill Dispenser	Group Medical Supply, LLC
PBA^c^ 001	MedQ Smart PillBox	Med-Q
PBA 002	MedGlider System 1 with Talking Reminder	Medport
PBA 003	VitaCarry Advanced Pill Case	VitaCarry
PBA 004	e-pill Multi-Alarm Pocket XL	e-pill
PBA 005	100-Hour Pill Reminder	Aidapt
PBA 006	eNNOVEA Weekly Planner with Advanced Auto Reminder	eNNOVEA Medical, LLC
PBA 007	Pill Box with Digital instruction	Shenzhen LongShitong Technology Co LTD
PBA 008	MedCentre System	MedCenter Systems, LLC
SM^d^ 001	Spencer Automatic Pill Dispenser	Custom Health
SM 002	CpaX Connected Medication Adherence Packaging	Jones Healthcare Group
SM 003	EllieGrid Smart Pill Organizer	EllieGrid

a MAT: medication adherence technology.

b APD: automated pill dispenser.

c PBA: pill box with alarm.

d SM: smart device.

### Study Procedure

Upon enrollment, participants provided written informed consent. A structured assessment was then conducted to identify potential barriers to effective medication use, including physical limitations, cognitive challenges, vision and hearing impairments, motivational factors, and environmental influences. Validated tools were used for these assessments: (1) the Self-Medication Assessment Tool (SMAT) [38,39] to measure physical, cognitive, and vision-related barriers; (2) the Daily Living Tasks Dependent on Vision (DLTV) [40,41] for vision-specific challenges; (3) the Whisper Test [42,43] for hearing capabilities; (4) the Self-Efficacy for Appropriate Medication Use Scale (SEAMS) [44-47] to assess motivational factors; and the Martin and Park Environmental Demands (MPED) [48] questionnaire to evaluate environmental factors such as routine complexity and perceived busyness.

Following the barrier assessment, usability testing of the devices was conducted using the cognitive walkthrough method [49]. This is a method used in user interface design to understand how new users interact with a device by simulating the process of exploring its functionality for the first time [49]. It is a task-based approach that focuses on evaluating the device’s ease of use and learnability, particularly for users who may not have previous experience or knowledge of the device [49]. The objective is to identify usability issues that could hinder the user’s ability to complete tasks efficiently and effectively [30-32]. To conduct a cognitive walkthrough, a participant information sheet and an evaluation sheet were prepared. The participant information sheet outlines specific tasks for users to perform with the devices. These tasks are designed to cover a range of functions and features of various devices, ensuring a comprehensive assessment of their usability. The tasks ranged from basic setup procedures like inserting batteries and unlocking the device to more complex interactions, such as setting up the current time, filling a tray with weekly medication, setting alarms, locking the device, and removing medication after an alarm. The evaluation sheet documented each task’s outcome, marking them as unassisted success, assisted success, or failure. Additionally, the sheet recorded task errors, indicating whether multiple attempts were required for a successful completion or if tasks were accomplished on the first try, and the time spent to complete each task. The evaluation sheet was developed based on previous research and expertise in usability testing of similar devices. During the testing sessions, 5 researchers (BB, GE, SP, IH, and RS) were paired in groups of 2 to independently observe participants and simultaneously record task performance. All evaluators received training on the study protocol and data recording procedures to ensure consistency. Training consisted of an initial structured session led by the corresponding author (TP), which lasted 4 hours and introduced all evaluators to the cognitive walkthrough methodology, task procedures, and data recording processes. Mock cognitive walkthroughs were conducted between groups of 3 evaluators to practice observing device testing among participants and recording data. Furthermore, for evaluators who joined the research team after the initial training session, a one-on-one training session was provided by the first author (BB), followed by observation of at least 2 full testing sessions with participants before independently participating in data collection. Following each session, the researchers reviewed their observations and reached a consensus to ensure accuracy and consistency in the recorded data. A mock medication regimen was developed using placebo medications (eg, candy and inert tablets) to simulate common prescription drugs and dosing schedules. Further details about the regimen are provided in Multimedia Appendix 1.

### Outcome Measures

Various outcome measures used in this study, along with their descriptions and the equations used for calculation, are provided in Table 2.

**Table 2.** Outcome measures, descriptions, and equations.

Outcome measure	Description and equation
Task success rate (unassisted)	Assesses users’ ability to independently complete device-related tasks, reflecting usability and learnability [32]. Task outcomes were categorized as unassisted success, assisted success, or failure based on predefined success criteria [31]. $\begin{array}{l} \text{Task success rate unassisted only}\;(\%)=\frac{\text{Number of tasks completed unassisted}}{\text{Total number of tasks required to use the device}}\times 100 \end{array}$
Time on task	The total elapsed time required to complete a task, also referred to as task completion time [32]. Time was recorded from task start to completion using a smartphone clock application.
Efficiency	Measures how effectively users complete tasks by relating unassisted task success to time spent [32]. $\text{Efficiency}=\frac{\frac{\text{Number of tasks completed successfully without assistance}}{\text{Total number of tasks required to set up the device}}}{\text{Total time spent completing the setup steps (minutes)}}$
Error rate	Quantifies unintentional user actions or mistakes made during task performance [31]. Errors were classified as present if additional attempts were required or absent if completed on the first attempt. $\begin{array}{l} \text{Error rate}\;(\%)=\frac{\text{Number of errors encountered}}{\text{Total number of tasks required to use the device}}\times 100 \end{array}$

### Data Collection and Statistical Analysis

Data were securely stored and managed using the REDCap platform (REDCap 15.1.2; Vanderbilt University). Descriptive statistics were used to summarize the key variables, and Spearman correlation analyses were performed to examine correlations between outcome measures and predictor variables such as age, number of devices tested, cognitive, physical, vision (SMAT and DLTV), SEAMS, and MPED scores. Product-level outcomes were analyzed using population-averaged generalized estimating equation (GEE) models. The unit of analysis was the individual product tried by a participant (product-level observations), with participants specified as the clustering unit to account for repeated observations within individuals. Models assumed a Poisson distribution with a log link and used an unstructured working correlation structure to account for within-participant correlation. The response variables were the counts of unassisted successes and the counts of errors. To account for variation in the number of subtasks per product, an offset equal to the log of the number of subtasks per product was included, such that model estimates correspond to rates (or percentages) of successes and errors per subtask. Models were fit using the *glmee* function from the *glmtoolbox* R package (version 4.3.3; RStudio). The box plots were created using SAS 9.4 (Enterprise Edition; SAS Institute) on the SAS OnDemand for Academics platform.

### Ethical Considerations

This study received ethical approval from the University of Waterloo Office of Research Ethics (ORE #45203). Informed consent was obtained from all participants before study procedures were initiated.

## Results

### Participant Demographics

A total of 96 individuals participated in the study, with a mean age of 75.1 (SD 7.7; range 61‐95) years. Among them, 36 (37.5%) were male, and 60 (62.5%) were female. The majority (86/96, 89.6%) reported taking medications regularly. Educational backgrounds varied as follows: 27.1% (26/96) had advanced degrees, 28.1% (27/96) had earned a bachelor’s degree, 17.7% (17/96) held diplomas, 17.7% (17/96) had a high school education or lower, and 1% (1/96) had not completed high school or held trade certificates. Most participants (83/96, 86.5%) lived independently in their own homes, while 13.5% (13/96) resided in independent retirement facilities.

Cardiovascular conditions were the most prevalent (56/96, 58.3%), followed by metabolic or endocrine disorders (37/96, 38.5%), musculoskeletal conditions (26/96, 27.1%), and vision-related issues (18/96, 18.8%). Less common health conditions included renal or urogenital disorders (7/96, 7.3%), stroke (5/96, 5.2%), and hematological conditions (4/96, 4.2%). A small proportion (5/96, 5.2%) reported having no significant medical history. Medication management approaches varied: 44.8% (43/96) used pillboxes, 11.5% (11/96) relied on alarm beepers, 10.4% (10/96) used blister packs, 8.3% (8/96) depended on reminders from others, 2.1% (2/96) used calendars, and 3.1% (3/96) reported other methods.

### Barriers to Medication Self-Management

Cognitive challenges, defined as low to moderate scores on the SMAT, were observed in 21.9% (21/96) of participants. Physical barriers, also assessed using SMAT, were present in 38.5% (37/96) of participants. Vision-related barriers were also notable, with 24% (23/96) scoring low or moderately low on the SMAT vision scale, and 38.5% (37/96) reporting challenges in vision-dependent tasks as measured by the DLTV scale. Hearing impairments, identified through the Whisper Test, were found in 57.3% (55/96) of participants. Additionally, motivational and environmental barriers, assessed using the SEAMS and the MPED Questionnaire, were each reported by 35.4% (34/96) of participants.

More details on participant demographics and barrier characteristics are provided in Table 3.

**Table 3.** Demographics, clinical, and barrier characteristics of study participants (N=96).

Variable and category or description	Statistical value
Age (y), mean (SD; range)	75.1 (7.7; 61-95)
Sex, n (%)	
Male	36 (37.5)
Female	60 (62.5)
Medication use (currently taking medication), n (%)	86 (89.6)
Education level, n (%)	
Below high school	1 (1)
High school	24 (25)
Trade certificate or diploma	1 (1)
Nonuniversity diploma	17 (17.7)
Bachelor’s degree	27 (28.1)
Master’s, Doctoral, or Professional degree	26 (27.1)
Place of residence, n (%)	
Private home	83 (86.5)
Retirement home	13 (13.5)
Self-reported medical conditions, n (%)	
Cardiovascular	56 (58.3)
Metabolic or endocrine	37 (38.5)
Musculoskeletal	26 (27.1)
Eye-related	18 (18.8)
Oral or gastrointestinal	16 (16.7)
Neurological	12 (12.5)
Respiratory	10 (10.4)
Mental health	8 (8.3)
Renal or urogenital	7 (7.3)
Cancer or neoplasms	5 (5.2)
Stroke	5 (5.2)
Hematological	4 (4.2)
Congenital disorders	2 (2.1)
Inflammatory or immune	2 (2.1)
Injuries or accidents	2 (2.1)
Ear-related	1 (1)
Infection	1 (1)
Reproductive health	1 (1)
Skin	1 (1)
Other	1 (1)
None reported	5 (5.2)
Medication management strategies, n (%)	
Pillbox	43 (44.8)
Alarm or beeper	11 (11.5)
Blister pack	10 (10.4)
Reminder from another person	8 (8.3)
Medication calendar	2 (2.1)
Other	3 (3.1)
SMAT^a^, n (%)	
Cognitive barrier	
High (≥90%)	70 (72.9)
Relatively high (≥80%)	5 (5.2)
Moderate (70%‐80%)	7 (7.3)
Low (≤69% approximately)	14 (14.6)
Physical barrier	
High (≥90%)	59 (61.5)
Moderate (76%‐84%)	11 (11.5)
Low (≤75%)	26 (27.1)
Vision barrier	
High (≥90%)	73 (76)
Moderate (76%‐84%)	4 (4.2)
Low (≤75%)	19 (19.8)
Vision barrier (DLTV^b^; impairment present), n (%)	37 (38.5)
Hearing barrier (Whisper Test; bilateral impairment), n (%)	55 (57.3)
Motivational barrier (SEAMS^c^; low self-efficacy), n (%)	34 (35.4)
Environmental barrier (MPED^d^; barrier present), n (%)	34 (35.4)

a SMAT: Self-Medication Assessment Tool; participants categorized as having moderate or low scores were considered to have impairments in cognitive, physical, and vision domains.

b DLTV: Daily Living Tasks Dependent on Vision; total score <79 indicates the presence of a vision-related barrier (lower scores reflect greater impairment).

c SEAMS: Self-Efficacy for Appropriate Medication Use Scale; total score <40 indicates low self-efficacy (motivational barrier), with lower scores representing reduced confidence in medication use.

d MPED: Martin and Park Environmental Demands; an environmental barrier is indicated by a busyness subscale score ≥15 (higher scores reflect greater busyness) and/or a routine subscale score <16 (lower scores reflect less structured routines).

Distribution of product testing by participant impairment type is presented in Multimedia Appendix 2.

### Outcome Measures

#### Observed Usability Performance Across MATs

The following section presents observed usability outcomes across the MATs tested in this study, summarizing patterns in average success rate unassisted, total error rate, efficiency for unassisted task success, and total task time.

#### Average Task Success Rate Unassisted

**Figure 1.**
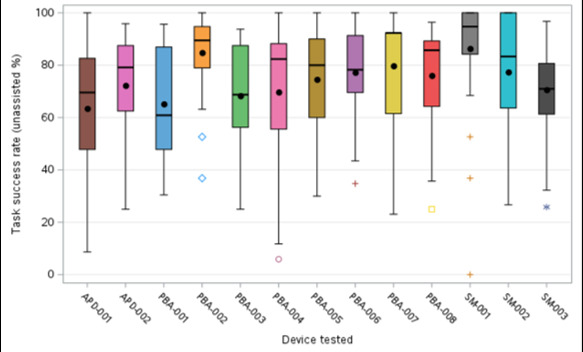
Unassisted task success rates of the various tested devices (boxes represent the IQR, horizontal lines indicate medians, dots indicate mean values, whiskers represent the spread of the data, and points represent outliers). APD: automated pill dispenser; PBA: pill box with alarm; SM: smart device.

The overall mean task success rate without assistance across all devices was 74.85% (SD 21.27), with a median of 80% (IQR 61.29-91.30), ranging from 0% to 100%. Smart pill dispensers, including the EllieGrid Smart Pill Organizer (SM-003; EllieGrid), Spencer Automatic Pill Dispenser (SM-001; Custom Health), and Jones Smart Blister Pack (SM-002; Jones Healthcare Group), generally showed higher success rates compared to electronic pill dispensers. Among the SMs, the Spencer Automatic Pill Dispenser (SM-001) had the highest mean success rate of 86.37% (SD 19.98), while the EllieGrid Smart Pill Organizer (SM-003) achieved 70.63% (SD 15.97). Electronic pill dispensers displayed more variability in performance, with success rates ranging from 63.48% to 84.82% (SD 26.42-15.21). The unassisted task success rates of the various tested devices are illustrated in Figure 1.

### Total Error Rate

**Figure 2.**
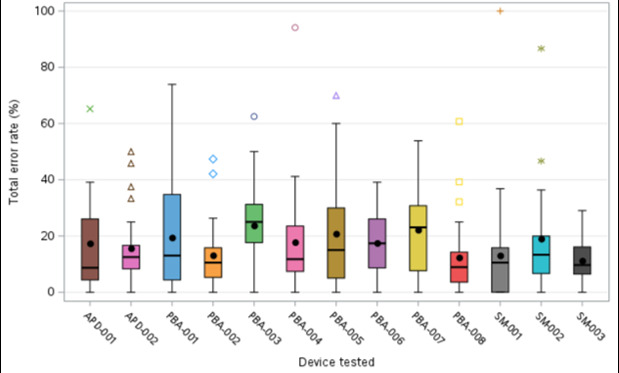
Total task error rates of the various tested devices (boxes represent the IQRs, horizontal lines indicate medians, dots indicate mean values, whiskers represent the spread of the data, and points represent outliers). APD: automated pill dispenser; PBA: pill box with alarm; SM: smart device.

The overall mean total error rate across all devices was 16.87% (SD 15.89), with a median of 12.7% (IQR 5.73-23.9), and it ranged from 0% to 100%, indicating considerable variability in user performance. The first quartile (Q1) was 5.73%, while the third quartile (Q3) reached 23.9%, showing that most error rates were clustered within this range. Among the products, the EllieGrid Smart Pill Organizer (SM-003) demonstrated the lowest mean error rate at 11.23% (SD 8.02), with a median of 9.68% (IQR 6.45-16.13), while the highest error rate was observed for the VitaCarry Advanced Pill Case (PBA-003; eNNOVEA Medical, LLC) at 23.76% (SD 14.38). Smart pill dispensers, such as the Spencer Automatic Pill Dispenser (SM-001) and Jones Smart Blister Pack (SM-002), had mixed performance, with Spencer showing a lower mean error rate of 13.09% (SD 18.07) compared with Jones at 19.06% (SD 19.39). The unassisted task error rates of the various tested devices are illustrated in Figure 2.

### Efficiency for Unassisted Task Success

**Figure 3.**
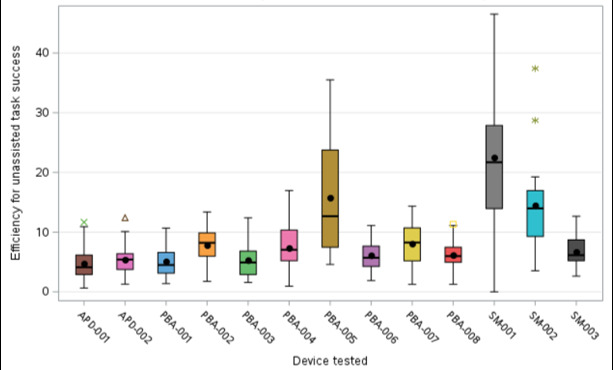
Efficiency for unassisted task success of the various tested devices (boxes represent the IQR, horizontal lines indicate medians, dots indicate mean values, whiskers represent the spread of the data, and points represent outliers). APD: automated pill dispenser; PBA: pill box with alarm; SM: smart device.

The overall efficiency for unassisted task success across all devices had a mean of 9.27 (SD 7.87), with a median of 6.83 (IQR 4.67-10.92), and values ranging from 0 to 46.51, indicating significant variability in performance. The highest efficiency unassisted was observed in the Spencer Automatic Pill Dispenser (SM-001), with a mean of 22.51 (SD 11.37) and a median of 21.70 (IQR 14.02-27.15), suggesting that SMs generally outperform electronic dispensers in task efficiency. Among electronic devices, the 100-Hour Pill Reminder (PBA-005; Aidapt) demonstrated relatively high efficiency, with a mean of 15.77 (SD 9.51), while devices such as the MedReady 1700 automated medication dispenser (APD-001; MedReady Inc) had lower efficiency, averaging 4.74 (SD 3.03). Smart pill dispensers, including the Jones Smart Blister Pack (SM-002), also performed well, with a mean efficiency of 14.48 (SD 7.5). The overall efficiency for unassisted task success of the various tested devices is illustrated in Figure 3.

### Total Task Time

**Figure 4.**
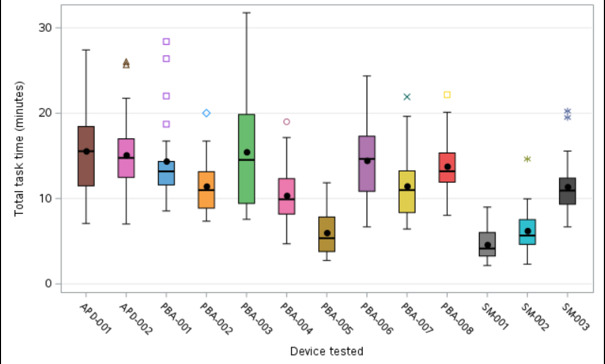
Total task time of the various tested devices (boxes represent the IQR, horizontal lines indicate medians, dots indicate mean values, whiskers represent the spread of the data, and points represent outliers). APD: automated pill dispenser; PBA: pill box with alarm; SM: smart device.

The overall total task time across all devices had a mean of 11.42 (SD 5.48) minutes, with a median of 10.94 (IQR 7.67-14.67) minutes, and ranged from 2.15 to 31.77 minutes, indicating variation in the time required to complete tasks. Smart pill dispensers, such as the Spencer Automatic Pill Dispenser (SM-001) and the Jones Smart Blister Pack (SM-002), demonstrated the shortest task times, with averages of 4.61 (SD 1.83) minutes and 6.24 (SD 2.65) minutes, respectively, possibly suggesting their ease of use and efficiency. In contrast, electronic pill dispensers, such as the MedReady 1700 Automated Medication Dispenser (APD-001), had longer task times, with an average of 15.58 (SD 5.22) minutes, indicating potentially more timely interactions required for operation. Among electronic dispensers, the 100-Hour Pill Reminder (PBA-005) had the shortest average task time at 6.01 (SD 2.64) minutes, showing relatively quicker use. The overall total task time of the various tested devices is illustrated in Figure 4.

Multimedia Appendix 3 provides more details on various outcome measures based on the devices tested and the barriers assessed. Although the data have been presented according to individual impairment categories, these findings should be interpreted carefully, as most participants had multiple coexisting impairments, making it difficult to isolate the effect of any single impairment category. Therefore, these subgroup comparisons are not fully generalizable.

### Significant Predictors of Performance-Based Usability Outcomes

Because participants tested varying numbers of devices and devices were not evaluated an equal number of times across participants, inferential analyses were conducted using Poisson GEE models with a log link, which account for within-participant correlation. This approach accounts for repeated measures within participants, unequal numbers of observations per participant, and variability in device usage, making it well-suited for analyzing correlated outcome data.

The selection of primary outcomes for regression analysis was informed by Spearman correlation analyses examining associations among outcome and predictor variables (Multimedia Appendix 4). Average unassisted task success rate and total error rate were selected as the primary modeled outcomes because they demonstrated meaningful associations with participant characteristics while representing distinct and interpretable dimensions of usability performance. Efficiency for unassisted task success and total task time were not modeled as primary outcomes, as these measures were highly correlated with task success rate and with each other, reflecting overlapping underlying constructs.

### Significant Predictors of Average Task Success Rate Unassisted

Poisson GEE model with a log link and within-participant correlation identified several device types and participant characteristics as statistically significant predictors (*P*<.05).

Relative to the reference device (APD-001), success rates were not statistically significantly different for APD-001 (*P*=.47) and PBA-001 (*P*=.82). In contrast, several automated pill dispensers and pill boxes demonstrated higher success rates, ranging from 23.5% (PBA-006; *P*=.009) to 528.5% (PBA-005; *P*<.001). One automated pill dispenser (PBA-008) performed with significantly lower success rates (−28.7%; *P*<.001) in comparison to APD-001. Among SMs, 2 (SM-001 and SM-002) performed with significantly higher success rates (97.2% increase, *P*<.001, and 212.4% increase, *P*<.001, respectively). SM-003 had significantly lower success rates (−39.9%, *P*<.001). Regarding participant characteristics, females had a significantly lower success rate than males (−9.3%, *P*=.002). Higher cognitive (1% increase per unit, *P*<.001), physical (3.8% increase per unit, *P*<.001), and vision scores (3.0% increase per unit, *P*<.001) were all associated with increased success rates. Similarly, higher SEAMS scores were associated with a 1.1% increase in success rate (*P*<.001), while MPED busyness score showed a marginally significant 0.8% increase in success rate (*P*=.05).

Table 4 shows the results of the final Poisson GEE model, showing the estimated effects of device type and participant characteristics on unassisted task success rates.

**Table 4.** Fixed effects estimates for the final model predicting average task success rate.

Predictor	Estimate^a^, standardized β coefficient (95% CI^b^)	SE	*z* score	% change^c^ (95% CI^d^)	*P* value^e^
APD^f^-001 (reference)	−3.743 (−4.243 to −3.243)	0.255	−14.678	—^g^	—
APD-002	0.056 (−0.096 to 0.208)	0.078	0.720	5.7% (−9.2% to 23.1%)	.47
PBA^h^-001	−0.020 (−0.196 to 0.155)	0.090	−0.228	−2% (−17.8% to 16.8%)	.82
PBA-002	0.663 (0.510 to 0.816)	0.078	8.497	94.1% (66.6% to 126.2%)	<.001
PBA-003	0.784 (0.641 to 0.926)	0.073	10.786	118.9% (89.9% to 152.4%)	<.001
PBA-004	0.715 (0.496 to 0.933)	0.111	6.413	104.4% (64.3% to 154.3%)	<.001
PBA-005	1.838 (1.690 to 1.987)	0.076	24.271	528.5% (441.8% to 629.1%)	<.001
PBA-006	0.211 (0.052 to 0.369)	0.081	2.607	23.5% (5.4% to 44.7%)	.009
PBA-007	1.358 (1.196 to 1.519)	0.082	16.472	288.8% (230.8% to 357%)	<.001
PBA-008	−0.338 (−0.483 to −0.194)	0.074	−4.593	−28.7% (−38.3% to −17.6%)	<.001
SM^i^-001	0.679 (0.529 to 0.829)	0.077	8.866	97.2% (69.7% to 129.2%)	<.001
SM-002	1.139 (0.944 to 1.335)	0.100	11.425	212.4% (156.9% to 279.8%)	<.001
SM-003	−0.509 (−0.678 to −0.340)	0.086	−5.900	−39.9% (−49.2% to −28.8%)	<.001
Sex (female vs male)	−0.098 (−0.161 to −0.035)	0.032	−3.043	−9.3% (−14.9% to −3.4%)	.002
Cognitive score	0.010 (0.005 to 0.015)	0.003	3.763	1% (0.5% to 1.6%)	<.001
Physical score	0.037 (0.021 to 0.053)	0.008	4.420	3.8% (2.1% to 5.5%)	<.001
Vision score	0.030 (0.014 to 0.046)	0.008	3.624	3% (1.4% to 4.7%)	<.001
SEAMS^j^ score (motivation)	0.011 (0.004 to 0.017)	0.003	3.314	1.1% (0.4% to 1.7%)	<.001
MPED^k^ busyness score (environmental)	0.008 (0.000 to 0.015)	0.004	1.969	0.8% (0% to 1.5%)	.049

a Estimates (standardized β coefficient) are presented on the log scale from a generalized estimating equations Poisson model with a log link and an unstructured correlation structure. For categorical variables (product tested), estimates represent comparisons relative to the reference category (APD-001).

b 95% CI for standardized β coefficient were calculated as β±1.96×SE. Percentage change in the expected error rate was computed as (e^β−1)×100.

c For continuous variables (eg, age and physical score), percentage change reflects the expected change in error rate per 1-unit increase in the predictor.

d Corresponding 95% CIs for percentage change were derived by exponentiating the confidence limits of β, calculated as [(e^(β−1.96×SE)−1)×100, (e^(β+1.96×SE)−1)×100].

e Statistical significance was assessed at *P*<.05.

f APD: automated pill dispenser.

g Not applicable.

h PBA: pill box with alarm.

i SM: smart device.

j SEAMS: Self-Efficacy for Appropriate Medication Use Scale.

k MPED: Martin and Park Environmental Demands.

Model diagnostic checks indicated that the final Poisson GEE model fit the data well. The dispersion parameter (0.289) and deviance-to-degrees-of-freedom ratio (1.06) showed no overdispersion, while the Pearson chi-square statistic (*χ*²_328_=328, *P*=.49) confirmed an adequate fit. Residual diagnostics, including a normal Q-Q plot and residuals versus fitted values plot, showed no major deviations or heteroscedasticity. The diagnostic plots are given in Multimedia Appendix 5.

### Significant Predictors of Total Error Rate

Poisson GEE model with a log link and within-participant correlation identified several device types and participant characteristics as statistically significant predictors of total error rate. Relative to APD-001, most devices did not differ significantly in error rates (*P*≥.24). However, PBA-008 showed a moderately significant lower error rate (−32.6%, *P*=.04). Among SMs, SM-001 showed a nonsignificant trend toward lower error rates (−35.9%, *P*=.08), while SM-002 and SM-003 did not differ significantly from the reference.

Regarding participant characteristics, increasing age was associated with a small but statistically significant increase in error rate (1.9% per unit increase, *P*=.02). Female participants had a significantly higher error rate compared with males (846.2% increase, *P*<.001). Higher physical scores were associated with increased error rates (46.1% increase per unit, *P*<.001), whereas higher vision scores were associated with reduced error rates (−9.9% per unit, *P*=.004). The DLTV summary score was also positively associated with error rate (2.2% increase per unit, *P*=.01). MPED routine score showed a marginally significant association with decreased error rate (−3.7%, *P*=.05). Additionally, there was a significant interaction between sex and physical score (−16.5%, *P*=.004), indicating that the effect of physical score on error rate differed by sex.

Table 5 presents the results of the final Poisson GEE model, showing the estimated effects of device type and participant characteristics on total error rate.

Model diagnostics indicated no evidence of overdispersion (overdispersion ratio=0.96) and an adequate fit (Pearson *χ*²_328_=328, *P*=.49). Residual diagnostics (normal Q-Q plot and residual vs fitted plot) revealed no major violations of model assumptions. The diagnostic plots are given in Multimedia Appendix 4.

**Table 5.** Fixed effects estimates for the final model predicting total error rate.

Predictor	Estimate, standardized β coefficient^a^ (95% CI^b^)	*z* score	% change^c^ (95% CI^d^)	*P* value^e^
APD^f^-001 (Ref)	−7.493 (−10.112 to −4.874)	−5.607	—^g^	—
APD-002	−0.015 (−0.418 to 0.387)	−0.075	−1.5% (−34.2% to 47.3%)	.94
PBA^h^-001	0.116 (−0.341 to 0.573)	0.496	12.3% (−28.9% to 77.3%)	.62
PBA-002	−0.282 (−0.751 to 0.187)	−1.178	−24.6% (−52.8% to 20.6%)	.24
PBA-003	0.138 (−0.184 to 0.459)	0.839	14.7% (−16.8% to 58.3%)	.40
PBA-004	0.210 (−0.204 to 0.625)	0.995	23.4% (−18.4% to 86.7%)	.32
PBA-005	0.069 (−0.386 to 0.525)	0.299	7.2% (−32% to 69%)	.76
PBA-006	−0.172 (−0.547 to 0.204)	−0.895	−15.8% (−42.2% to 22.7%)	.37
PBA-007	0.193 (−0.166 to 0.553)	1.053	21.3% (−15.3% to 73.8%)	.29
PBA-008	−0.395 (−0.780 to −0.009)	−2.006	−32.6% (−54.2% to −0.9%)	.04
SM^i^-001	−0.444 (−0.933 to 0.045)	−1.779	−35.9% (−60.7% to 4.6%)	.08
SM-002	0.058 (−0.452 to 0.568)	0.221	5.9% (−36.4% to 76.4%)	.82
SM-003	−0.171 (−0.537 to 0.196)	−0.914	−15.7% (−41.6% to 21.6%)	.36
Age	0.018 (0.003 to 0.034)	2.315	1.9% (0.3% to 3.5%)	.02
Sex (Female)	2.247 (0.926 to 3.568)	3.334	846.2% (152.4% to 3446.3%)	<.001
Physical score	0.379 (0.166 to 0.592)	3.491	46.1% (18.1% to 80.8%)	<.001
Vision score	−0.104 (−0.175 to −0.034)	−2.920	−9.9% (−16% to −3.4%)	.004
DLTV^j^ summary score (vision)	0.021 (0.005 to 0.038)	2.536	2.2% (0.5% to 3.9%)	.01
MPED^k^ routine score	−0.038 (−0.075 to 0.000)	−1.981	−3.7% (−7.3% to 0%)	.05
Sex×physical score	−0.181 (−0.303 to −0.058)	−2.896	−16.5% (−26.2% to −5.7%)	.004

a Estimates (standardized β coefficient) are presented on the log scale from a generalized estimating equations Poisson model with a log link and an unstructured correlation structure. For categorical variables (product tested), estimates represent comparisons relative to the reference category (APD-001).

b 95% CI for standardized β coefficient were calculated as β±1.96×SE. Percentage change in the expected error rate was computed as (e^β−1)×100.

c For continuous variables (eg, age and physical score), percentage change reflects the expected change in error rate per one-unit increase in the predictor.

d Corresponding 95% CIs for percentage change were derived by exponentiating the confidence limits of β, calculated as [(e^(β−1.96×SE)−1)×100, (e^(β+1.96×SE)−1)×100].

e Statistical significance was assessed at *P*<.05.

f APD: automated pill dispenser.

g Not applicable.

h PBA: pill box with alarm.

i SM: smart device.

j DLTV: Daily Living Tasks Dependent on Vision.

k MPED: Martin and Park Environmental Demands.

## Discussion

### Principal Findings

This study tested the usability and UX of 13 MATs among older adults with diverse cognitive, physical, sensory, motivational, and environmental capabilities. By focusing on performance-based usability metrics, such as unassisted task success rate, error rate, task completion time, and efficiency, this research extends previous MAT usability literature by examining not only device-level variability but also how individual user characteristics interact with device design during first-time use. These results are intended to provide an overview of how users interacted with a range of commercially available devices under the study conditions, rather than to rank devices or establish definitive performance comparisons. The devices included vary widely in features, functionality, and required task complexity, reflecting real-world options rather than standardized experimental comparators. Additionally, participants tested differing numbers of devices, and devices were not evaluated an equal number of times across participants.

A key finding was the variability in usability across MATs, regardless of device category or intended function. This indicates that usability cannot be inferred solely from whether a device is “smart” or “electronic,” but instead reflects specific design features, interaction complexity, and task demands. These findings align with previous work by Patel et al [27], which reported wide variability in unassisted task success, task time, and workload across electronic medication adherence products, even when devices served similar purposes. This study extends this literature by demonstrating that such variability persists among newer-generation smart MATs and users with diverse functional profiles.

The observed variability reinforces the importance of performance-based usability testing. Although subjective measures such as the SUS capture user perceptions, previous research has shown discrepancies between perceived usability and objective task performance. Consistent with this, participants in this study often rated devices as acceptable despite frequent errors or difficulty completing setup tasks. More broadly, usability research has demonstrated weak correlations between subjective ratings and objective performance measures, particularly for complex or safety-critical technologies [50]. Collectively, these findings highlight the value of performance-based metrics for identifying usability risks that may not be captured through self-report alone, especially for MATs where interaction errors may have clinically significant consequences such as medication-related harm.

Regression analyses showed that cognitive, physical, visual, and motivational (SEAMS) scores were significant predictors of unassisted task success, consistent with previous literature on technology use among older adults [27,29]. Cognitive ability has been consistently linked to successful MAT use, with studies of automated dispensers and reminder systems reporting marked reductions in unassisted success among individuals with lower MMSE scores, even when impairment is mild [29]. Vision also emerged as a significant predictor, with both SMAT vision and DLTV scores associated with usability outcomes, echoing evidence that many technologies impose high visual demands and that inadequate accommodation of vision-specific barriers can compromise safe and effective use among older adults [51,52]. Motivational self-efficacy was likewise associated with higher success rates, consistent with findings that individuals with greater confidence are more likely to persist, implement effective strategies, and achieve better outcomes [53,54].

In contrast, most devices did not differ significantly from the reference device in error-rate models, with only 1 device (PBA 008) showing a statistically significant reduction. This apparent inconsistency reflects an important methodological distinction between task failure and task error. Errors were recorded only when tasks were completed with incorrect actions or multiple attempts, whereas failures were not coded as errors. As a result, devices with lower success rates may also show lower error rates simply because users were unable to progress far enough to generate observable errors. Conversely, devices that allowed task completion often did so with frequent missteps, yielding higher error rates despite greater success. From a medication safety perspective, successful task completion accompanied by repeated errors may pose greater real-world risk than early task abandonment. Similar conclusions have been reported in usability and human-factors research, which emphasizes that usability metrics represent distinct but interdependent constructs and should not be interpreted alone [50].

Some predictors of error rate were less intuitive. Higher physical scores were associated with increased error rates, likely because participants with better physical ability progressed further and interacted more extensively with device features, creating more opportunities for observable errors. Similarly, the positive association between DLTV scores and error rates likely reflects greater task exposure rather than poorer vision, as participants with better vision advanced into more complex interactions. These insights underscore the importance of interpreting error rates alongside task success and engagement measures rather than as standalone indicators [50].

Sex differences were evident across usability outcomes, with female participants demonstrating lower unassisted task success, higher error rates, and a significant interaction between sex and physical ability. These results suggest that sex may moderate how older adults interact with MATs, potentially reflecting differences in confidence, experience, and interaction strategies shaped by historical gender roles [55]. Although gender-related factors were not directly assessed, the consistency of sex effects across success and error outcomes highlights the need for sex-sensitive testing and inclusive design approaches to support safe and effective MAT use among older adults.

All devices were evaluated under untrained, first-use laboratory conditions to approximate real-world initial use. While appropriate for assessing early usability and learnability, laboratory testing cannot capture adaptive behaviors, learning over time, or caregiver involvement that may emerge during long-term home use. Evidence from longitudinal studies indicates that some older adults become more proficient and confident with technology with continued exposure [56]. Accordingly, the performance-based outcomes reported here should be interpreted as indicators of initial usability rather than long-term usability or continued adoption in everyday settings.

### Study Strengths, Limitations, and Future Research Directions

A key strength of this study is the inclusion of older adults with multiple barriers, including motivational and environmental challenges, and the evaluation of both smart and electronic MATs. While this study provides valuable insights, several limitations should be acknowledged. Although the inclusion of participants with multiple coexisting impairments improves the real-world application, it may limit the ability to draw conclusions about the effects of a single impairment on the usability of a specific device. Future studies using more controlled designs, such as focusing on participants with a single type of impairment and testing a smaller number of devices, may help provide clearer device-specific conclusions. However, such designs may be less feasible in real-world aging populations, as most older adults experience a combination of impairments rather than a single isolated condition. The sample primarily consisted of Canadian English-speaking older adults, which may limit the generalizability of the findings to non-English speakers who might encounter additional language-related barriers when using MATs. In addition, the initial sample size estimation was guided by Green’s formula based on 6 primary impairment domains. However, the final Poisson GEE models included a broader set of predictors, including demographic variables. In addition, variability in the number of observations per device may result in an uneven distribution of data across predictors. While the overall sample size was adequate, these factors may introduce some risk of overfitting and affect the stability of model estimates.

Future research should explore the usability of these technologies in more diverse populations, including individuals with limited health literacy or linguistic diversity, as well as how device design and instructional materials can be optimized to support individuals with different and multiple barriers in safely and successfully completing tasks. Additionally, longitudinal research is needed to examine the long-term impact of MATs on medication adherence and health outcomes. Understanding how long-term use of these technologies influences adherence behaviors over time can provide valuable insights for health care providers and policymakers. Future studies should also explore how older adults interact with these technologies in real-world settings, including their reliance on instructional materials, learning approaches, and help-seeking behaviors. Moreover, this study only collected information on participants’ sex. Future research should include both sex and gender measures to better capture sociocultural and biological influences on technology use.

### Conclusion

This study highlights the role of performance-based usability testing in identifying the factors for successful use of MATs among older adults with diverse capabilities. By assessing unassisted task success rates, error rates, task completion times, and efficiency, the findings demonstrate substantial variability in usability across MATs, reflecting differences in device design, task complexity, and interaction demands rather than inherent device superiority.

Cognitive, physical, visual, motivational, and environmental factors were significant predictors of performance, highlighting that successful MAT use depends not only on technological capabilities but also on alignment with age-related abilities and confidence in medication management. The presence of higher error rates even among users who successfully completed tasks further highlights that usability and safety cannot be interpreted from task completion alone. Observed sex differences in performance reinforce the need for inclusive, user-centered design and evaluation approaches that account for diverse interaction styles and experiential backgrounds.

## Supplementary material

10.2196/88398 Multimedia Appendix 1 Mock medication regimen.

10.2196/88398 Multimedia Appendix 2 Distribution of product testing by participant impairment type.

10.2196/88398 Multimedia Appendix 3 Descriptive statistics for average task success rate (unassisted), total error rate, efficiency (unassisted), and total task time across participant subgroups and product types.

10.2196/88398 Multimedia Appendix 4 Spearman correlation.

10.2196/88398 Multimedia Appendix 5 Model diagnostic plots.

## Abbreviations

APD: automated medication dispenser
DLTV: Daily Living Tasks Dependent on Vision
GEE: generalized estimating equation
MAT: medication adherence technology
MMSE: Mini-Mental State Examination
MPED: Martin and Park Environmental Demands
SEAMS: Self-Efficacy for Appropriate Medication Use Scale
SM: smart device
SMAT: Self-Medication Assessment Tool
SUS: System Usability Scale
USE: Usefulness, Satisfaction, and Ease of Use
UX: user experience
