# Supplementary material for: Performance-Based Usability of Medication Adherence Technologies Among Older Adults With Diverse Capabilities: Quantitative Study
Source: JMIR Aging. 2026 Jul 13;9:e88398. doi: 10.2196/88398 (PMC13361894; doi:10.2196/88398)
Supplement: Multimedia Appendix 1 [file aging-v9-e88398-s001.docx]

**Mock Medication regimen**

A simulated medication regimen was designed for this study to replicate the complexity of real-life medication schedules often managed by older adults. Developed in-house, this mock regimen aimed to assess the usability of technologies by mimicking the organization of medications in every-day of MATs while ensuring safety by substituting actual medications with placebo tablets, placebo capsules, and candy.

The mock regimen included placebo equivalents for common medications used to manage chronic conditions, structured as follows:

- **Warfarin:** 2 mg once daily on Monday, Wednesday, and Friday, and 3 mg once daily on Tuesday, Thursday, Saturday, and Sunday.
- **Pantoprazole:** 20 mg taken twice daily.
- **Phenytoin:** 100 mg, with one capsule in the morning and two capsules in the evening.
- **Propranolol:** 20 mg, with half a tablet once daily for the first two days, followed by one full tablet daily.
