# Supplementary material for: Performance-Based Usability of Medication Adherence Technologies Among Older Adults With Diverse Capabilities: Quantitative Study
Source: JMIR Aging. 2026 Jul 13;9:e88398. doi: 10.2196/88398 (PMC13361894; doi:10.2196/88398)
Supplement: Multimedia Appendix 2 [file aging-v9-e88398-s002.docx]

Distribution of Product Testing by Participant Impairment Type

| Product | Total Tested | Cognitive | Physical | Vision_SMAT | Vision_DLTV | Motivation | Environmental | Hearing |
| --- | --- | --- | --- | --- | --- | --- | --- | --- |
| APD-001 | 25 | 6 | 16 | 5 | 9 | 12 | 6 | 11 |
| APD-002 | 29 | 6 | 10 | 5 | 11 | 9 | 13 | 16 |
| PBA-001 | 23 | 5 | 9 | 5 | 7 | 5 | 8 | 14 |
| PBA-002 | 26 | 5 | 11 | 5 | 8 | 5 | 7 | 12 |
| PBA-003 | 26 | 6 | 7 | 5 | 10 | 10 | 11 | 15 |
| PBA-004 | 23 | 5 | 8 | 5 | 9 | 9 | 8 | 12 |
| PBA-005 | 24 | 5 | 8 | 5 | 10 | 9 | 8 | 18 |
| PBA-006 | 25 | 6 | 7 | 5 | 11 | 8 | 10 | 15 |
| PBA-007 | 27 | 7 | 5 | 5 | 11 | 9 | 13 | 19 |
| PBA-008 | 30 | 5 | 9 | 6 | 9 | 10 | 12 | 20 |
| SM-001 | 39 | 8 | 15 | 5 | 13 | 10 | 13 | 26 |
| SM-002 | 22 | 5 | 12 | 5 | 8 | 8 | 8 | 14 |
| SM-003 | 29 | 6 | 12 | 6 | 10 | 11 | 9 | 12 |
