# Supplementary material for: Performance-Based Usability of Medication Adherence Technologies Among Older Adults With Diverse Capabilities: Quantitative Study
Source: JMIR Aging. 2026 Jul 13;9:e88398. doi: 10.2196/88398 (PMC13361894; doi:10.2196/88398)
Supplement: Multimedia Appendix 3 [file aging-v9-e88398-s003.docx]

Descriptive statistics for average task success rate (unassisted), total error rate, efficiency (unassisted), and total task time across participant subgroups and product types

|  | Average Task Success Rate Unassisted | | | | | | | | Total Error Rate | | | | | | | | |
| --- | --- | --- | --- | --- | --- | --- | --- | --- | --- | --- | --- | --- | --- | --- | --- | --- | --- |
|  | **Overall** | **Cog** | **Phy** | **Vis (S)** | **Vis(D)** | **Hear** | **Moti** | **Envi** | **Overall** | **Cog** | **Phy** | **Vis (S)** | **Vis(D)** | **Hear** | **Moti** | **Envi** |  |
| N | 348 | 75 | 129 | 67 | 126 | 204 | 115 | 126 | 348 | 75 | 129 | 67 | 126 | 204 | 115 | 126 |  |
| Mean | 74.85 | 64.86 | 65.24 | 61.87 | 69.3 | 74.11 | 67.96 | 75.76 | 16.87 | 21.44 | 16.52 | 20.08 | 15.31 | 17.04 | 18.16 | 14.81 |  |
| Median | 80 | 68.42 | 67.74 | 63.64 | 75.74 | 79.58 | 70 | 87.3 | 12.7 | 17.86 | 10.53 | 16.13 | 10.26 | 12.5 | 12.5 | 11.76 |  |
| Std_Dev | 21.27 | 24.36 | 24.37 | 25.81 | 24.03 | 21.45 | 24.88 | 22.75 | 15.89 | 18 | 16.89 | 21.26 | 17.1 | 16.33 | 19.93 | 12.49 |  |
| Min | 0 | 0 | 0 | 0 | 0 | 0 | 0 | 13.04 | 0 | 0 | 0 | 0 | 0 | 0 | 0 | 0 |  |
| Max | 100 | 100 | 100 | 100 | 100 | 100 | 100 | 100 | 100 | 100 | 100 | 100 | 100 | 100 | 100 | 53.85 |  |
| Q1 | 61.29 | 51.32 | 47.83 | 43.48 | 53.85 | 60 | 53.1 | 60.87 | 5.73 | 9.35 | 5.26 | 4.26 | 4.35 | 5.73 | 5.26 | 5.26 |  |
| Q3 | 91.3 | 83.77 | 86.67 | 83.48 | 89.47 | 91.3 | 89.29 | 92.31 | 23.9 | 27.85 | 22.58 | 27.86 | 21.66 | 23.9 | 25 | 20.62 |  |
| **Product** | **N** | **Mean** | **Median** | **Std_Dev** | **Min** | **Max** | **Q1** | **Q3** | **N** | **Mean** | **Median** | **Std_Dev** | **Min** | **Max** | **Q1** | **Q3** |  |
| APD-001 | 25 | 63.48 | 69.57 | 26.42 | 8.7 | 100 | 47.83 | 82.61 | 25 | 17.39 | 8.7 | 15.93 | 0 | 65.22 | 4.35 | 26.09 |  |
| APD-002 | 29 | 72.27 | 79.17 | 19.2 | 25 | 95.83 | 62.5 | 87.5 | 29 | 15.66 | 12.5 | 12.73 | 0 | 50 | 8.33 | 16.67 |  |
| PBA-001 | 23 | 65.22 | 60.87 | 19.84 | 30.43 | 95.65 | 47.83 | 82.61 | 23 | 19.47 | 13.04 | 18.63 | 0 | 73.91 | 6.52 | 30.43 |  |
| PBA-002 | 26 | 84.82 | 89.47 | 15.21 | 36.84 | 100 | 80.26 | 94.74 | 26 | 13.16 | 10.53 | 11.65 | 0 | 47.37 | 5.26 | 15.79 |  |
| PBA-003 | 26 | 68.34 | 68.75 | 18.71 | 25 | 93.75 | 56.25 | 85.94 | 26 | 23.76 | 25 | 14.38 | 0 | 62.5 | 17.92 | 29.69 |  |
| PBA-004 | 23 | 69.76 | 82.35 | 27.78 | 5.88 | 100 | 61.11 | 88.24 | 23 | 17.82 | 11.76 | 19.49 | 0 | 94.12 | 7.87 | 20.59 |  |
| PBA-005 | 24 | 74.58 | 80 | 20.85 | 30 | 100 | 60 | 90 | 24 | 20.83 | 15 | 21.65 | 0 | 70 | 7.5 | 30 |  |
| PBA-006 | 25 | 77.26 | 78.26 | 17.49 | 34.78 | 100 | 69.57 | 91.3 | 25 | 17.53 | 17.39 | 9.58 | 0 | 39.13 | 8.7 | 26.09 |  |
| PBA-007 | 27 | 79.77 | 92.31 | 21.46 | 23.08 | 100 | 65.38 | 92.31 | 27 | 22.22 | 23.08 | 16.57 | 0 | 53.85 | 7.69 | 30.77 |  |
| PBA-008 | 30 | 76.07 | 85.71 | 20.68 | 25 | 96.43 | 64.29 | 89.29 | 30 | 12.38 | 8.93 | 13.16 | 0 | 60.71 | 3.57 | 14.29 |  |
| SM-001 | 39 | 86.37 | 94.74 | 19.98 | 0 | 100 | 84.21 | 100 | 39 | 13.09 | 10.53 | 18.07 | 0 | 100 | 2.63 | 13.16 |  |
| SM-002 | 22 | 77.41 | 83.33 | 20.93 | 26.67 | 100 | 64.39 | 98.33 | 22 | 19.06 | 13.33 | 19.39 | 0 | 86.67 | 6.67 | 20 |  |
| SM-003 | 29 | 70.63 | 70.97 | 15.97 | 25.81 | 96.77 | 61.29 | 80.65 | 29 | 11.23 | 9.68 | 8.02 | 0 | 29.03 | 6.45 | 16.13 |  |
| Cog – Cognitive Barrier  Phy - Physical Barrier  Vis (S) – Vision barrier measured using SMAT  Vis (D) – Vision barrier measured using DLTV  Hear – Hearing Barrier  Moti – Motivational Barrier  Envi – Environmental Barrier | | | | | | | | | | | | | | | | |  |

|  | Efficiency for Unassisted | | | | | | | | Total Task Time (minutes) | | | | | | | | |
| --- | --- | --- | --- | --- | --- | --- | --- | --- | --- | --- | --- | --- | --- | --- | --- | --- | --- |
|  | **Overall** | **Cog** | **Phy** | **Vis (S)** | **Vis(D)** | **Hear** | **Moti** | **Envi** | **Overall** | **Cog** | **Phy** | **Vis (S)** | **Vis(D)** | **Hear** | **Moti** | **Envi** |  |
| N | 348 | 75 | 129 | 67 | 126 | 204 | 115 | 126 | 348 | 75 | 129 | 67 | 126 | 204 | 115 | 126 |  |
| Mean | 9.27 | 7.21 | 7.81 | 7.4 | 8.19 | 9.61 | 7.92 | 9.73 | 11.42 | 12.71 | 12.42 | 11.98 | 11.99 | 11.49 | 11.92 | 11.14 |  |
| Median | 6.83 | 5.39 | 5.36 | 5.2 | 6.13 | 7.01 | 5.98 | 7.57 | 10.94 | 11.85 | 11.85 | 10.92 | 10.97 | 10.7 | 11 | 9.64 |  |
| Std_Dev | 7.87 | 6.92 | 7.87 | 6.91 | 7.02 | 8.04 | 7.56 | 8.42 | 5.48 | 6.1 | 5.96 | 6.36 | 6.15 | 6.18 | 5.8 | 5.4 |  |
| Min | 0 | 0 | 0 | 0 | 0 | 0 | 0 | 0.74 | 2.15 | 2.22 | 2.18 | 2.32 | 2.32 | 2.22 | 2.15 | 2.15 |  |
| Max | 46.51 | 42.74 | 43.39 | 37.41 | 37.41 | 43.17 | 46.51 | 46.51 | 31.77 | 31.77 | 27.42 | 31.77 | 31.77 | 31.77 | 31.77 | 24.37 |  |
| Q1 | 4.67 | 3.45 | 3.11 | 2.81 | 3.36 | 4.47 | 3.73 | 4.65 | 7.67 | 8.54 | 8.22 | 7.58 | 7.52 | 6.63 | 8.12 | 7.54 |  |
| Q3 | 10.92 | 8.33 | 9.28 | 10.19 | 10.34 | 11.64 | 9.5 | 11.16 | 14.67 | 15.82 | 16.18 | 16.06 | 15.99 | 15.29 | 14.68 | 14.74 |  |
| **Product** | **N** | **Mean** | **Median** | **Std_Dev** | **Min** | **Max** | **Q1** | **Q3** | **N** | **Mean** | **Median** | **Std_Dev** | **Min** | **Max** | **Q1** | **Q3** |  |
| APD-001 | 25 | 4.74 | 4.12 | 3.03 | 0.63 | 11.66 | 2.89 | 6.15 | 25 | 15.58 | 15.55 | 5.22 | 7.08 | 27.42 | 11.48 | 18.43 |  |
| APD-002 | 29 | 5.39 | 5.41 | 2.45 | 1.28 | 12.42 | 3.75 | 6.38 | 29 | 15.12 | 14.77 | 4.31 | 7.02 | 26.03 | 12.48 | 17 |  |
| PBA-001 | 23 | 5.11 | 4.5 | 2.49 | 1.38 | 10.68 | 3.14 | 6.57 | 23 | 14.39 | 13.18 | 5.08 | 8.55 | 28.4 | 11.67 | 14.18 |  |
| PBA-002 | 26 | 7.82 | 8.21 | 2.89 | 1.76 | 13.36 | 5.98 | 9.88 | 26 | 11.46 | 10.99 | 3.06 | 7.35 | 20.02 | 8.92 | 13.09 |  |
| PBA-003 | 26 | 5.31 | 4.92 | 2.93 | 1.57 | 12.39 | 2.93 | 6.64 | 26 | 15.47 | 14.53 | 6.49 | 7.57 | 31.77 | 9.47 | 19.72 |  |
| PBA-004 | 23 | 7.34 | 7.05 | 4.04 | 0.95 | 16.97 | 5.25 | 9.62 | 23 | 10.37 | 9.92 | 3.52 | 4.7 | 19 | 8.2 | 12.01 |  |
| PBA-005 | 24 | 15.77 | 12.66 | 9.51 | 4.59 | 35.5 | 7.65 | 23.66 | 24 | 6.01 | 5.36 | 2.64 | 2.75 | 11.85 | 3.8 | 7.76 |  |
| PBA-006 | 25 | 6.12 | 5.71 | 2.72 | 1.88 | 11.11 | 4.26 | 7.66 | 25 | 14.46 | 14.65 | 5.08 | 6.68 | 24.37 | 10.85 | 17.3 |  |
| PBA-007 | 27 | 8.07 | 8.25 | 3.75 | 1.25 | 14.35 | 5.2 | 10.52 | 27 | 11.47 | 11 | 3.96 | 6.43 | 21.92 | 8.64 | 13.11 |  |
| PBA-008 | 30 | 6.16 | 5.98 | 2.51 | 1.26 | 11.32 | 4.95 | 7.45 | 30 | 13.79 | 13.2 | 3.53 | 8.03 | 22.17 | 11.93 | 15.31 |  |
| SM-001 | 39 | 22.51 | 21.7 | 11.37 | 0 | 46.51 | 14.02 | 27.15 | 39 | 4.61 | 4.15 | 1.83 | 2.15 | 9 | 3.33 | 5.76 |  |
| SM-002 | 22 | 14.48 | 13.98 | 7.5 | 3.54 | 37.41 | 9.7 | 16.9 | 22 | 6.24 | 5.67 | 2.65 | 2.32 | 14.63 | 4.65 | 7.35 |  |
| SM-003 | 29 | 6.7 | 6.14 | 2.55 | 2.63 | 12.66 | 5.2 | 8.7 | 29 | 11.39 | 10.93 | 3.3 | 6.68 | 20.25 | 9.33 | 12.4 |  |

*(Note: The descriptive statistics presented for each device are intended to provide an overview of observed usability outcomes across the MATs tested in this study, including average unassisted success rate, total error rate, efficiency unassisted, and total task time. These results reflect observed findings under controlled study conditions using commercially available devices and are not intended to demonstrate the superiority of any device. They should not be interpreted as definitive comparisons of device performance in real-world settings. The devices vary in features, functionality, and task complexity, and participants tested differing numbers of devices. As such, these summaries do not account for the underlying data structure, including repeated observations within individuals and variation in testing frequency.)*
