# Supplementary material for: Performance-Based Usability of Medication Adherence Technologies Among Older Adults With Diverse Capabilities: Quantitative Study
Source: JMIR Aging. 2026 Jul 13;9:e88398. doi: 10.2196/88398 (PMC13361894; doi:10.2196/88398)
Supplement: Multimedia Appendix 4 [file aging-v9-e88398-s004.docx]

Spearman correlation between outcome and predictor variables

| Variables | Age | Cognition Score | Physical Score | Vision SMAT Score | Vision DLTV Score | SEAMS Score (Motivation) | MPED Busyness Score | MPED Routine Score | Number of Subtask per Device | Number device tested | Average success rate Unassisted | Total Error Rate | Efficiency Unassisted | Total Time on Task |
| --- | --- | --- | --- | --- | --- | --- | --- | --- | --- | --- | --- | --- | --- | --- |
| Age | 1 | 0 | 0 | 0 | 0 | 0 | 0 | 0 | 0 | 0 | 0 | 0 | 0 | 0 |
| Cognition Score | -0.126 | 1 | 0 | 0 | 0 | 0 | 0 | 0 | 0 | 0 | 0 | 0 | 0 | 0 |
| Physical Score | -0.274 | 0.166 | 1 | 0 | 0 | 0 | 0 | 0 | 0 | 0 | 0 | 0 | 0 | 0 |
| Vision SMAT Score | -0.135 | 0.39 | 0.144 | 1 | 0 | 0 | 0 | 0 | 0 | 0 | 0 | 0 | 0 | 0 |
| Vision DLTV Score | -0.086 | 0.302 | 0.138 | 0.535 | 1 | 0 | 0 | 0 | 0 | 0 | 0 | 0 | 0 | 0 |
| SEAMS Score (Motivation) | 0.134 | 0.05 | 0.079 | 0.174 | 0.161 | 1 | 0 | 0 | 0 | 0 | 0 | 0 | 0 | 0 |
| MPED Busyness Score | -0.316 | 0.053 | 0.148 | 0.001 | -0.156 | -0.12 | 1 | 0 | 0 | 0 | 0 | 0 | 0 | 0 |
| MPED Routine Score | -0.011 | 0.079 | -0.015 | 0.134 | 0.315 | 0.275 | -0.183 | 1 | 0 | 0 | 0 | 0 | 0 | 0 |
| Number of Subtask per Device | -0.164 | 0.05 | -0.164 | -0.04 | 0.053 | -0.072 | -0.008 | 0.134 | 1 | 0 | 0 | 0 | 0 | 0 |
| Number device tested | 0.002 | 0.126 | 0.142 | 0.347 | 0.259 | 0.213 | 0.109 | 0.023 | -0.249 | 1 | 0 | 0 | 0 | 0 |
| Average success rate Unassisted | -0.232 | 0.359 | 0.437 | 0.411 | 0.353 | 0.335 | 0.079 | 0.126 | 0.011 | 0.34 | 1 | 0 | 0 | 0 |
| Total Error Rate | 0.133 | -0.101 | 0.105 | -0.107 | 0.157 | -0.046 | -0.047 | -0.073 | -0.153 | 0.058 | -0.262 | 1 | 0 | 0 |
| Efficiency Unassisted | -0.203 | 0.274 | 0.341 | 0.313 | 0.272 | 0.335 | 0.005 | 0.067 | -0.256 | 0.308 | 0.724 | -0.279 | 1 | 0 |
| Total Time on Task | 0.206 | -0.071 | -0.151 | -0.1 | -0.061 | -0.153 | -0.011 | 0.178 | 0.313 | -0.164 | -0.287 | 0.154 | -0.752 | 1 |

**Key Points**

- **Age** shows weak to moderate negative correlations with several outcome measures:

Negative correlations with **Physical Score** (-0.274), **MPED Busyness Score** (-0.316), and **Average Success Rate Unassisted** (-0.232).

A positive correlation with **Total Time on Task** (0.206), suggesting older participants may take longer on tasks.

- **Cognition Score** is positively correlated with:

**Vision SMAT Score** (0.390) and **Average Success Rate Unassisted** (0.359).

Also positively related to **Efficiency Unassisted** (0.274), indicating higher cognitive ability may contribute to better task performance.

- **Physical Score** has meaningful positive correlations with:

**Average Success Rate Unassisted** (0.437) and **Efficiency Unassisted** (0.341), and a negative correlation with **Total Time on Task** (-0.151).

This implies better physical ability may improve success and reduce task time.

- **Vision Scores (SMAT and DLTV)** are moderately correlated with each other (0.535), and positively associated with:

**Success Rate** (0.411 and 0.353, respectively).

**Efficiency** (0.313 and 0.272), reinforcing the importance of visual function in unassisted task performance.

- **Motivation (SEAMS Score)** is positively associated with:

**Success Rate** (0.335) and **Efficiency** (0.335), suggesting motivational factors are meaningful for task outcomes.
