# Supplementary material for: Performance-Based Usability of Medication Adherence Technologies Among Older Adults With Diverse Capabilities: Quantitative Study
Source: JMIR Aging. 2026 Jul 13;9:e88398. doi: 10.2196/88398 (PMC13361894; doi:10.2196/88398)

**Model Diagnostic Plots for average task success rate unassisted and total error rate**

**Figure S1.** Normal Q-Q plot of deviance residuals for the final Poisson GEE model - **average task success rate unassisted**


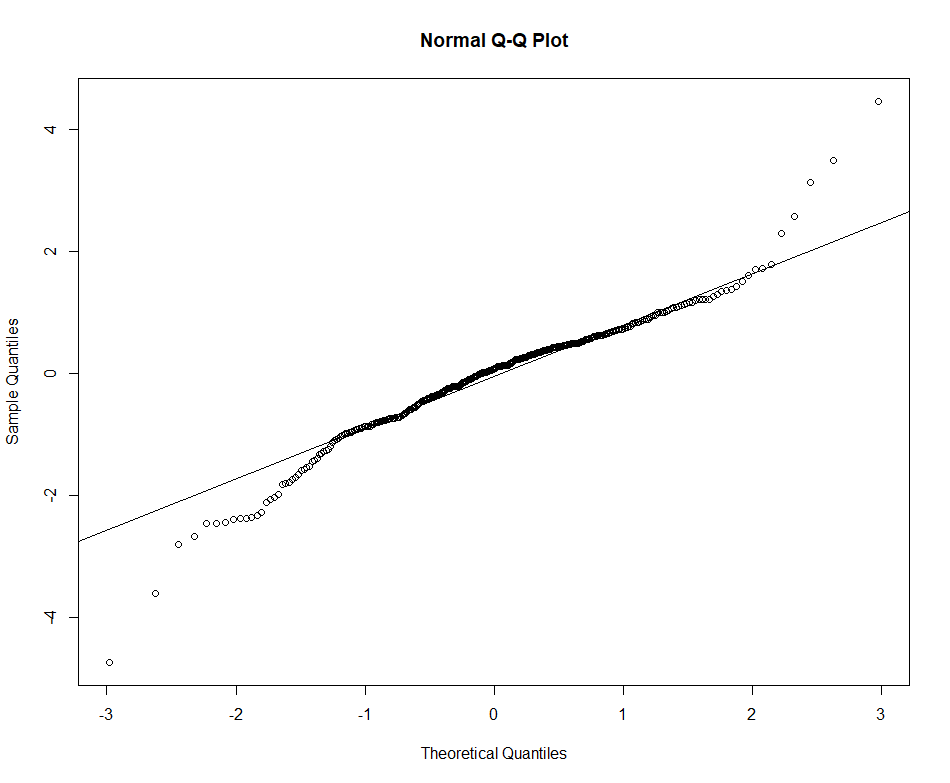


Footnote: The Q-Q plot compares the distribution of deviance residuals to a theoretical normal distribution. Points closely follow the diagonal reference line, indicating that the residuals approximate normality. Minor deviations at the tails are expected but do not suggest serious violations of model assumptions.

**Figure S2.** Residuals versus fitted values plot for the final Poisson GEE model - **average task success rate unassisted**


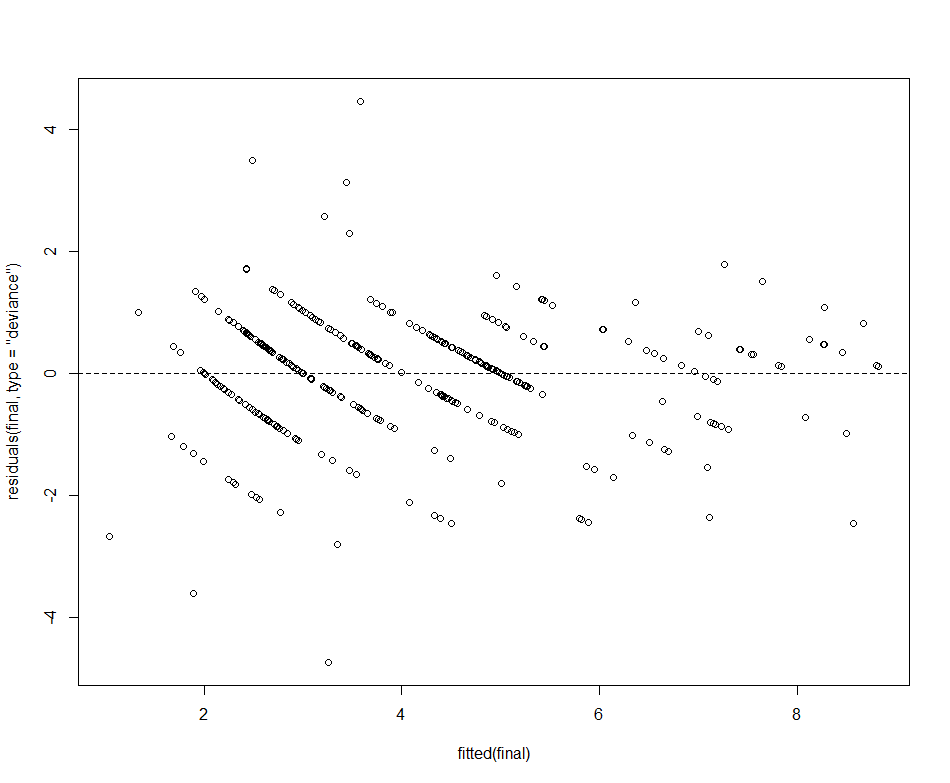


Footnote: The plot shows deviance residuals against the fitted values from the model. The absence of a clear pattern or systematic trend indicates that the mean structure of the model is appropriate. The residuals are symmetrically scattered around zero, suggesting no major heteroscedasticity or misspecification issues.

**Figure S3.** Normal Q-Q plot of deviance residuals for the final Poisson GEE model – **total error rate**


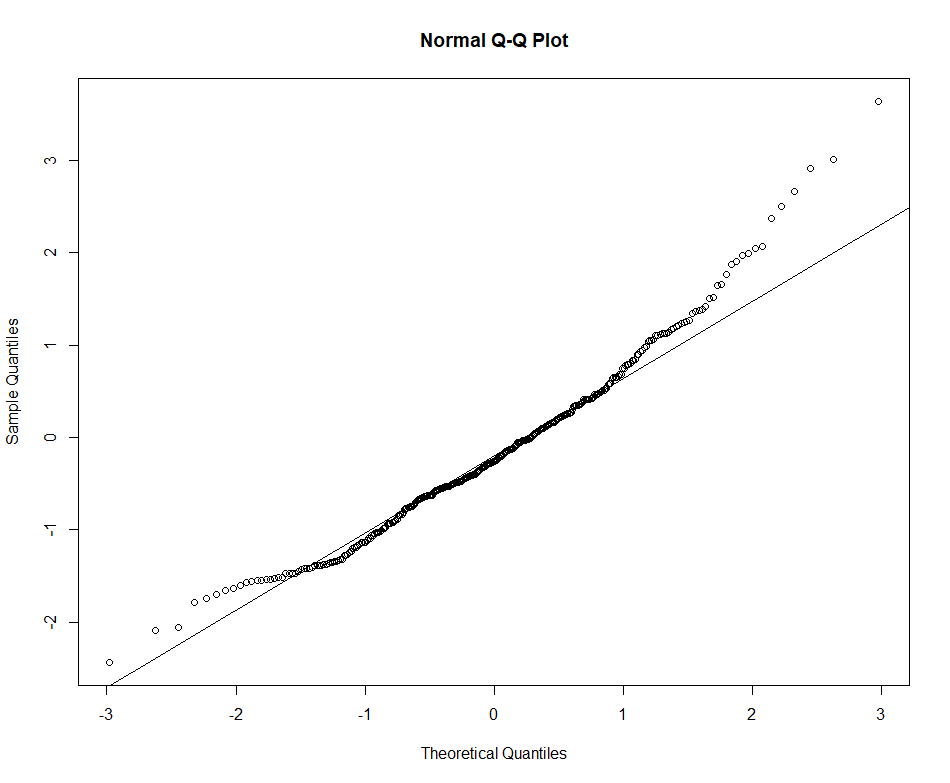


**Figure S4.** Residuals versus fitted values plot for the final Poisson GEE model - **total error rate**


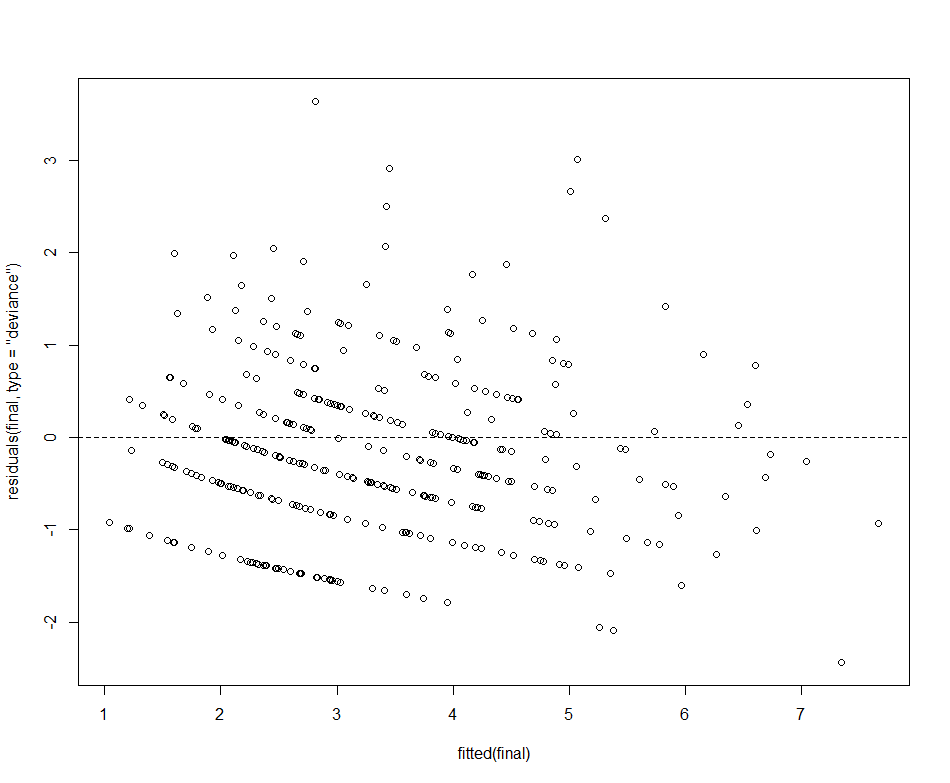

Supplement: Multimedia Appendix 5 [file aging-v9-e88398-s005.docx]
